# Maternal age, autistic-like traits and mentalizing as predictors of child autistic-like traits in a population-based cohort

**DOI:** 10.1186/s13229-022-00507-4

**Published:** 2022-06-15

**Authors:** Novika Purnama Sari, Pauline W. Jansen, Laura M. E. Blanken, Amber N. V. Ruigrok, Peter Prinzie, Henning Tiemeier, Simon Baron-Cohen, Marinus H. van IJzendoorn, Tonya White

**Affiliations:** 1 Department Psychology, Education and Child Studies, Erasmus University Rotterdam, Rotterdam, The Netherlands; 2 Department of Child and Adolescent Psychiatry/Psychology, Erasmus University Medical Centre, Rotterdam, The Netherlands; 3 Generation R Study Group, Erasmus University Medical Center Rotterdam, Rotterdam, The Netherlands; 4 Department of Radiology and Nuclear Medicine, Erasmus University Medical Centre, Rotterdam, The Netherlands; 5 Department of Psychiatry, Amsterdam University Medical Centre, University of Amsterdam, Amsterdam, The Netherlands; 6 Department of Social and Behavioral Sciences, Harvard T.H. Chan School of Public Health, Boston, MA USA; 7 Research Department of Clinical Educational and Health Psychology, UCL, University of London, London, UK; 8 Autism Research Centre, Department of Psychiatry, University of Cambridge, Cambridge, UK

**Keywords:** Maternal age, Mentalizing, Autistic-like traits, Children

## Abstract

**Background:**

Many empirical studies suggest that higher maternal age increases the likelihood of having an autistic child. However, little is known about factors that may explain this relationship or if higher maternal age is related to the number of autistic-like traits in offspring. One possibility is that mothers who have a higher number of autistic-like traits, including greater challenges performing mentalizing skills, are delayed in finding a partner. The goal of our study is to assess the relationship between maternal age, mentalizing skills and autistic-like traits as independent predictors of the number of autistic-like traits in offspring.

**Methods:**

In a population-based study in the Netherlands, information on maternal age was collected during pre- and perinatal enrolment. Maternal mentalizing skills and autistic-like traits were assessed using the Reading the Mind in the Eyes Test and the Autism Spectrum Quotient, respectively. Autistic-like traits in children were assessed with the Social Responsiveness Scale. A total of 5718 mother/child dyads had complete data (*M*_*agechild*_ = 13.5 years; 50.2% girls).

**Results:**

The relationship between maternal age and autistic-like traits in offspring best fits a U-shaped curve. Furthermore, higher levels of autistic features in mothers are linked to higher levels of autistic-like traits in their children. Lower mentalizing performance in mothers is linked to higher levels of autistic-like traits in their children.

**Limitations:**

We were able to collect data on both autistic-like traits and the mentalizing skills test in a large population of mothers, but we did not collect these data in a large number of the fathers.

**Conclusions:**

The relationships between older and younger mothers may have comparable underlying mechanisms, but it is also possible that the tails of the U-shaped curve are influenced by disparate mechanisms.

**Supplementary Information:**

The online version contains supplementary material available at 10.1186/s13229-022-00507-4.

## Introduction

Autism spectrum disorder (henceforth, autism) is a neurodevelopmental condition that indicates differences in both social communication and social interactions, coupled with focused interests and repetitive patterns of behavior [1–3]. We use the term ‘autism’ to avoid the potentially stigmatizing effect of the word ‘disorder’ and to acknowledge that autism is both a disability and an example of neurodiversity [4]. Not only is autism highly heritable, but autistic-like traits also show moderate to high heritability [5–7]. Furthermore, a recent study has provided new evidence that variation in autistic-like traits has been found to be linked to clinical diagnoses of autism [7–12]. Autistic-like traits can often be measured by 18 months of age [13–16] and autism is typically diagnosed prior to five years of age, although diagnosis in practice can occur in later childhood, adolescence or even adulthood [17, 18]. Approximately 60% to 90% of the variation in autism is explained by genetic factors. The remaining variance may result from other factors, including those present in prenatal, perinatal and postnatal environments [19–21].

Advanced maternal and paternal age may also increase the likelihood of having a child with autism or higher levels of autistic-like traits [22–29]. Croen et al., [22]  reported that the relative likelihood of autism increased by 1.31 times with each 10-year increase in maternal age. Likewise, another study [30] reported that children of fathers 50 years old and older were 2.2 times more likely to have a diagnosis of autism compared to children of fathers under 30 years old. These studies provide support that factors related to advanced maternal and paternal age increase the likelihood for autism. However, the factors associated with this link are unclear. Since the emergence of autism has a large genetic component, one possibility is that parents with more autistic-like traits, and thus greater genetic loading for autism, take more time to navigate the social nuances involved in mating [31, 32]. If this was the case, parents of children with autism should have a higher number autistic-like traits and characteristics associated with autism, such as impaired theory of mind [33].

Theory of Mind (ToM), also known as mentalizing or cognitive empathy, refers to the ability to reflect on mental states in oneself and others [34, 35]. Studies support a positive association between mothers’ mentalizing skills and the mentalizing skills in their children [36]. The positive association, however, is weak and not always replicated in other studies. The association between maternal and child ToM abilities could be via genetic transmission, as empathy is partly heritable [37]. Alternatively, mothers who are skilled in predicting and interpreting their child’s behavior may be a role model for their children, who in turn develop their own abilities to interpret the behavior of others [38]. A twin study by [39] demonstrated a high contribution from both shared and non-shared environments in ToM performance in 5-year-old children.

Since many studies have only examined the relationship between maternal age and autism or autistic-like traits in children, little is known about how the mother’s level of mentalizing skills and autistic-like traits contributes to this association. Mothers who have higher levels of autistic-like traits or who show lower mentalizing skills may have children at a later age, perhaps in part due to the social challenges of forming intimate relationships. Therefore, the aim of our study was to use a large, population-based study of child development to first replicate the finding that increasing maternal and paternal age at conception predict higher levels of children’s autistic-like traits. Second, we aimed to explore whether higher levels of maternal autistic-like traits and lower levels of maternal mentalizing skills are related to higher levels of autistic-like traits in offspring. Finally, we examined whether maternal mentalizing skills and maternal autistic-like traits partly explain the association between maternal age and child autistic-like traits.

## Method

### Design and study participants

The study was conducted as part of the Generation R Study, an ongoing population-based cohort of mothers and children in Rotterdam, the Netherlands [40]. Pregnant women were enrolled between 2002 and 2006 and were followed longitudinally. In our earlier work, we showed that in spite of some attrition, the Generation R cohort continues to reflect the general multiethnic population of Rotterdam [41]. We performed non-response analyses comparing demographic information at different waves of data collection to the initial recruitment into the Generation R Study. For the 9-to-11 and 13-to-15-year-old follow-up waves, consent for maternal and child data was available for 6706 children. Participants with missing data on children’s autistic-like traits (*N* = 988) were excluded, resulting in 5718 participants with complete data. Table 1 presents the baseline characteristics of the study sample and Additional file 1: Fig S1 shows a flowchart of participant inclusion.

**Table 1:** Participant characteristics

Study characteristics	*N*	Mean (SD)
*Maternal*		
Age at conception	5718	30.9 (4.7)
Education (%)		
High	3172	55.5
Mid	1520	26.6
Low	532	10.2
Autistic-like traits score	3610	50.9 (8.9)
Mentalizing skill score	3993	17.6 (3.4)
*Paternal*		
Age at conception	4936	33.5 (5.5)
Education (%)		
High	2933	51.3
Mid	1230	21.5
Low	666	11.6
*Child*		
Age (years)	4700	13.5 (0.4)
Sex (% boys)	2849	49.8
*National origin (%)*		
Dutch	3622	63.3
Moroccan, Turkish, Cape Verdean, Antillean, Surinamese	1176	20.6
Other western	528	9.2
Other non-western	349	6.1
Autistic-like traits score	5718	0.3 (0.2)

Data represent means (SDs) unless specified otherwise

Missing data: 8.6% maternal education, 13.7% paternal age, 15.5% paternal education, 17.9% child age, 0.8% child national origin, 30.1% maternal mentalizing skills, 36.8% maternal autistic traits

### Measures

#### Maternal and paternal age

The age of the mothers and fathers was obtained at the time of recruitment into the study. Since gestational age of the child at the time of enrolment differed slightly between parents, we calculated parental age at conception by subtracting the gestational age at birth of the child from the parental age at enrolment.

#### Child autistic-like traits

When children were between 5-to-6 and 13-to-15 years-of-age, an 18-item abbreviated form of the Social Responsiveness Scale (SRS) was administered to the parents of all participating children to obtain a quantitative measure of autistic-like traits [42, 43]. The validity of the abbreviated SRS has been described in previous studies [43, 44]. This short form consists items of social communication, social cognition, and social mannerism. The SRS short-form highly correlates (*r* = 0.93) with the full 65 item version [6, 43]. The SRS had high stability over time in a longitudinal study with 1–5 years of follow up (test–retest correlation = 0.90) [45, 46]. With the SRS, parents (in most cases the mother) rated their child’s behaviour over the past six months. Individual item scores for the SRS were summed and weighted by the number of items completed. Higher total scores indicate a greater number of social difficulties with scores ranging from 0 to 51. For main analyses of our study, we used SRS scores that were reported when the children were 13-to-15 years-of-age. Cronbach’s alpha indicated adequate reliability for the SRS 5/6 (*α* = 0.92) and SRS 13/15 (*α* = 0.71). The SRS 5/6 showed a relatively strong correlation with the SRS 13/15 (*r* = 0.54, *p* < 0.001).”

Multiple-gating procedure was used to clinically diagnose children with autism. Details were described in other papers of Generation R Study [47, 48].

#### Maternal mentalizing skills

A computerised version of the 27-item abbreviated Reading the Mind in the Eyes Test (RMET) [41, 49] was administered to mothers during a visit at the research centre when the children were 9 years-of-age. We used the computerized abbreviated version of the RMET due to time constraints in administering the task to a large number of mothers at the research center. The task was explained to mothers in several introductory slides, which also comprised practice trials. In each trial, the mothers were presented with a photograph of the eye region of the face of different actors and actresses, and they were asked which of four words best represented what this person was feeling or thinking. Words were numbered from one to four and mothers responded by using the corresponding number on the keyboard. If they were unsure of the exact meaning of a word, mothers could click on that word and were provided a short definition of the word. An answer was required to move to the next trial and there was no time limit for answering the trials. To obtain a total score, the number of correctly answered trials were summed. In a pilot study of 214 mothers who took the complete 37-item version of the test, a correlation of 0.94 was demonstrated between the short version and the long version of the test (*p* < 0.001). Test–retest reliability was 0.63 [50]. Cronbach’s α for our sample was = 0.64.

#### Maternal autistic-like traits

We used the validated 28-item abbreviated version of the Autism Spectrum Quotient (AQ-28) [51] to obtain self-reports of autistic-like traits in adults. The questionnaire was mailed shortly after the centre visit, comprising descriptive statements assessing personal preferences and habits, to which participants respond on a 4-point Likert scale, with possible choices including: ‘1 = *definitely agree*’; ‘2 = *slightly agree*’; ‘3 = *slightly disagree*’ and ‘4 = *definitely disagree*’ (e.g., ‘*My attention is often drawn to car number plates, or similar sequences*’). Total scores were weighted for the number of items completed. The Cronbach alpha in this study was 0.81. Although we used different measures of autistic-like traits in mothers and children, the AQ and SRS have been shown to be strongly associated (*r* = 0.64) [52, 53].

### Covariates

Several sociodemographic variables (child age, sex, and national origin; and maternal educational level) were considered as covariates in the analyses, as these demographic variables have been reported to be linked with our outcome variables in previous studies [6, 54, 55] and may function as precision variables [56, 57]. We do note, however, that maternal age at the time of pregnancy may be influenced by demographic factors, such as pursuing higher education prior to pregnancy. Thus, by providing different models of analyses, the role of specific demographics can be assessed. Information on sex and age of the child was obtained from the medical records completed by community midwives and obstetricians at birth. The child’s national origin was classified by the countries of birth of the parents, according to the Dutch Standard Classification Criteria of Statistics Netherlands [58]. Maternal education, defined by the highest attained education, was divided into: low education, consisting of no education and primary school only; medium education, which included secondary school level; high education, including higher vocational training and university level.

### Statistical analyses

We used t-tests for continuous data or χ^2^ for categorical data to perform non-response analyses comparing demographic characteristics of participants included versus those not included, i.e., due to attrition or missing exposures or outcome variables. In line with previous studies (e.g., [59], a maximum of 25% missing items was allowed for all collected measures (i.e., child autistic-like traits, maternal autistic-like traits, and maternal mentalizing skills). For those measures with more than 75% items completed, a mean total score was calculated, which was weighted by the number of items completed*.* To check the nature relationship between maternal autistic-like traits, mentalizing skills and child autistic-like traits, we ran nonlinearity correlations using *devtools* and *nlcor* package in R. Results indicated that the nonlinearity correlations do not show significant improvement over the linear correlations (*r* maternal autistic-like traits: 0.1, *r* maternal mentalizing skills: 0.1), suggesting that the relationship is linear.

Linear regression analyses were performed to examine the relation between maternal age and child autistic-like traits. In models 1 and 2, we added maternal age, and the quadratic term for maternal age to model curvilinear relations. The quadratic term for maternal age was added to assess nonlinear associations with maternal age. In the third model, the sociodemographic variables were included as covariates (child age, sex, and national origin; maternal educational level). Finally, in the fourth model, we adjusted for maternal characteristics separately (maternal mentalizing skills (model 4^a^) or maternal autistic-like traits (model 4^b^) and assessed the changes in effect estimates of the maternal age. Furthermore, to assess the relationship between maternal autistic-like traits and mentalizing skills with child autistic-like traits, we also used linear regression analyses, adjusting for sociodemographic variables. Independent and dependent variables were standardized into z-scores for ease of comparison across the various models. For missing data on maternal education, paternal age, child national origin, maternal mentalizing skills, and maternal autistic-like traits, twenty imputed datasets were generated and pooled estimates were calculated using multiple imputation with chained equations (MICE) [60]. These data were considered missing completely at random before imputation according to Little’s MCAR test (χ^2^ (23) =  = 43.7, *p* = 0.08).

We performed the analysis for paternal age, for which missing data (13.7%) on paternal age were imputed. We only ran analyses for associations between paternal age and child autistic-like traits since there was very little data on paternal mentalizing skills and paternal autistic-like traits. In addition, given that the correlation between maternal and paternal age was 0.61, we re-ran the analyses adjusting for paternal age in the maternal analyses, and adjusting for maternal age in the paternal age analyses.

### Sensitivity analyses

In sensitivity analyses, we repeated the analyses of maternal age and paternal age with child autistic-like traits by using SRS at 5-to-6 years of age to assess whether similar quadratic patterns were found, compared to the SRS measured at 13 years-of-age. In addition, we performed a sensitivity analysis including only mothers who were pregnant with their first child (*N* = 5061) using SRS at 13 years-of-age, to account for the possibility that mothers with higher autistic-like traits may start families later. Finally, we ran a sensitivity analysis by categorizing age in three groups, corresponding to a previous meta-analysis [61] to offer the opportunity to compare our results with the prior meta-analysis. The primary comparison contrasted maternal and paternal age groups: comparing reference age group (25–29 years) with younger (≤ 20 years) and older (≥ 35 years) parental age by using linear regression. Covariates that were included were child age, sex, national origin, and parental educational level.

## Results

### Sample characteristics

Child and maternal characteristics are presented in Table 1. Autistic-like traits in children were assessed at a mean age of 13.5 years (*SD* = 0.4 years). Within our sample, 49.8% were boys and 63% of the children were of Dutch national origin. Of the mothers in our sample, 55.5% had a high educational level (higher vocational training or university level). The mean age of the mothers at child birth was 30.9 years (*SD* = 4.7 years) and fathers was 33.5 years (*SD* = 5.5 years). A correlation matrix between the variables is presented in Additional file 1: Table S2. The highest correlation between variables of interest was between maternal and paternal age (*r* = 0.61). Approximately 1.4% of the children in our study had received a clinical diagnosis of autism.

### Non-response analyses

The non-response analysis showed that the mothers included in the study were more likely to have higher educational levels (69.1% vs. 30.9%, χ^2^(2; 2548), *p* < 0.001) and to be older (30.9 (*SD* = 4.7) years vs. 28.3 (*SD* = 5.6) years, *t* (4183) = 24.1, *p* < 0.001) than non-responders. Children of mothers included in the study were more likely to be of Dutch national origin (64.4% vs. 35.6%, χ^2^(3, 2043), *p* < 0.001).

### Maternal mentalizing skills, maternal autistic-like traits, and child autistic-like traits

Results of regression analyses after adjusting for sociodemographic variables revealed that higher maternal mentalizing skills were found to be associated with less maternal autistic-like traits (*β* =  − 0.111, *R*^2^ = 0.090, 95% *CI* − 0.147;—0.074, *p* < 0.001). Lower maternal mentalizing skills were associated with higher child autistic-like traits (*β* = − 0.073, *R*^2^ = 0.055, 95% *CI* − 0.113; − 0.032, *p* < 0.001), see Table 2 Model 4^a^. Higher maternal autistic-like traits were associated with higher child autistic-like traits (*β* = 0.165, *R*^2^ = 0.074, 95% *CI* 0.127; 0.202, *p* < 0.001), see Table 2 Model 4^b^.

**Table 2:** Association between maternal age, maternal mentalizing skills, maternal autistic-like traits and child autistic-like traits (*N* = 5,718)

	Model 1	*R*^2^(change)	Model 2	*R*^2^(change)	Model 3	*R*^2^(change)	Model 4^a^	*R*^2^(change)	Model 4^b^	*R*^2^(change)
	β (95% CI)	*p*	*β* (95% CI)	*p*	*β* (95% CI)	*p*	*β* (95% CI)	*p*	*β* (95% CI)	*p*
Step 1		**(.011)**								
Maternal age	− .102 (− .130; − .073)	< .001	− .108 (− .136; − .079)	< .001	− .064 (− .094; − .034)	< .001	− .067 (− .097; − .037)	< .001	− .058 (− .088; − .028)	< .001
Step 2				**(.007)**						
Maternal age squared			.077 (.049; .105)	< .001	.052 (.024; .080)	< .001	.050 (.022; .079)	.001	.047 (.019; .075)	.001
Step 3						**(.032)**				
Child age					.035 (.007; .063)	.015	.034 (.006; .062)	.018	.039 (.010; .068)	.009
Child sex					− .209 (− .263; − .154)	< .001	− .209 (− .264; − .154)	< .001	− .211 (− .266; − .155)	< .001
Child national origin					.008 (.001; .015)	.034	.006 (− .001; .013)	.080	.003 (− .004; .010)	.452
Maternal education					− .126 (− .153; − .098)	< .001	− .110 (− .138; − .082)	< .001	− .092 (− .121; − .063)	< .001
Step 4^a^								**(.005)**		
Maternal mentalizing skills							− .073 (− .113; − .032)	.001	−	−
Step 4^b^										**(.024)**
Maternal autistic − like traits							−	−	.165 (.127; .202)	< .001

*R* squared change is calculated by subtracting the changed explained variance in the model with the explained variance of the previous model. ^a^Model adjusted with maternal mentalizing skills. ^b^Model adjusted with maternal autistic-like traits

*β* = standardized beta, 95% CI = 95% confidence interval. Bold denotes significant, *p* < 0.05)

### Maternal age, paternal age, and child autistic-like traits

**Fig. 1:**
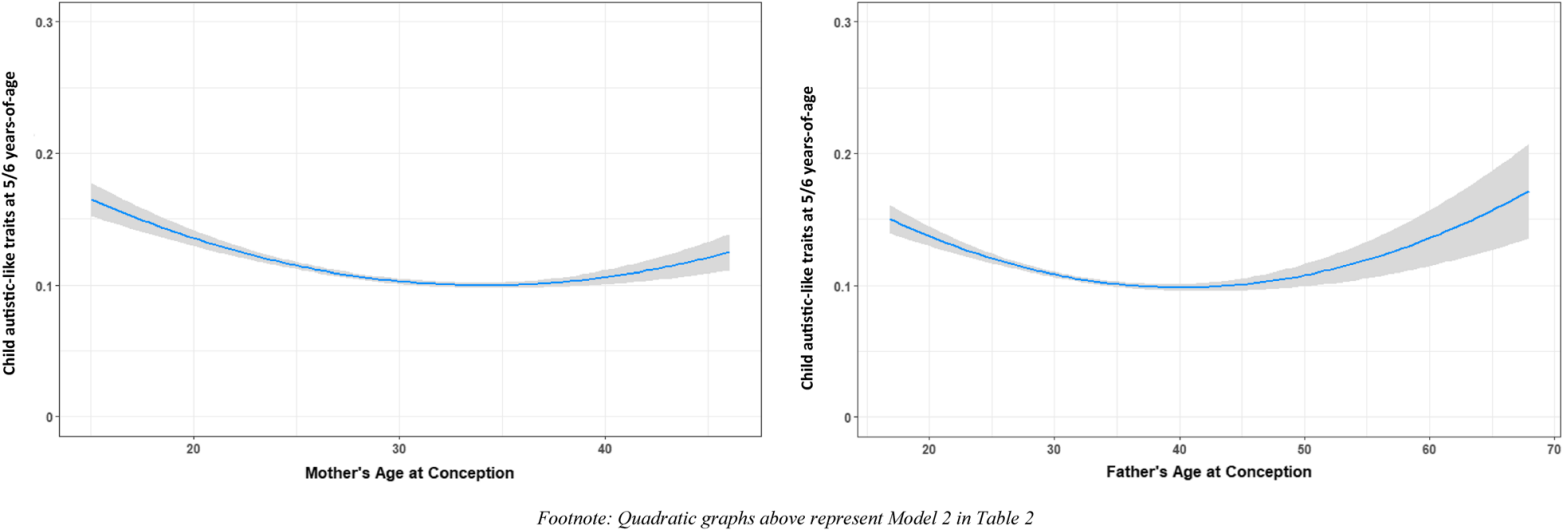
Quadratic graphs between parental age and child autistic-like traits at 13 years-of-age

In linear regression analyses with maternal age predicting autistic-like traits, a quadratic relationship reflected an optimal fit with higher order models not significantly improving the explained variance (*R*^2^ = 0.01773, *RMSE* = 0.066) compared to either a third-degree polynomial or linear splines (*R*^2^ = 0.01782, *RMSE* = 0.069; *R*^2^ = 0.01791, *RMSE* = 0.076, respectively), see Additional file 1: Figs S4 and S5. Adding the quadratic term of maternal age with child autistic-like traits resulted in a small yet significant change in explained variance over the linear model (*R*^2^ change_maternalagesquared_ = 0.007, *R*^2^ = 0.018, *p* < 0.001). The estimate of the first standardized coefficient (*β*_1 _= − 0.064) represents the downward linear trend in the values of Y along the X axis, and the value of the second standardized coefficient (*β*_*2 *_= 0.052) represents the curvature in the data. In addition, we checked the robustness of our standard error and additionally used the White Test to assess Heteroskedasticity. Results showed that our standard error was robust, with a small change of the confidence interval (e.g., *β*_*maternal age*_ =  − 0.064, 95% *CI* − 0.096; − 0.032, *p* < 0.001) and White Test for Heteroskedasticity was significant (*p* < 0.001), indicating a potential nonlinear relation between our predictors and the variance of the residuals. Furthermore, the association remained significant after adjusting for paternal age. Next, we examined the relation between paternal age and child autistic-like traits. A similar curvilinear pattern was found for paternal age (*R*^2^ change_paternalage_ = 0.002, *R*^2^ = 0.051, *p* < 0.001). However, after adjusting for maternal age, the association between paternal age and child autistic-like traits was no longer statistically significant. The nonlinear relation of maternal age and paternal age with autistic-like traits in children is presented in Fig. 1.

### Maternal age, maternal autistic-like traits, maternal mentalizing skills, and child autistic-like traits

The quadratic association between maternal age and child autistic-like traits remained significant after separately adjusting for maternal mentalizing skills or maternal autistic-like traits in model three. These analyses resulted in only an effect estimate decrease of 3.9% when adjusting for maternal mentalizing skills and a 9.6% decrease when adjusting for maternal autistic-like traits. Maternal autistic-like traits predicted child autistic-like traits more strongly (*β*_adjusted_ = 0.165, 95% *CI* 0.127; 0.202 *p* < 0.001) compared to maternal mentalizing skills.

Additionally, we run an analysis by adding both maternal autistic-like traits and mentalizing skills in one model and the results remained significant (*R squared change* = 0.029).

### Sensitivity analyses

We conducted sensitivity analyses by using SRS score at 5-to-6 years of age to assess the association between maternal age and paternal age with child autistic-like traits. These analyses showed a similar pattern (i.e., a U-shaped curve). In addition, we performed sensitivity analyses including only mothers with a parity of first pregnancy (*N* = 5061), and this also showed similar results as in the total study population (*N* = 5781). The quadratic term of maternal age with child autistic-like traits resulted in a significant, yet small change in explained variance and this effect remained significant after adjusting for maternal mentalizing skills or maternal autistic-like traits.

In addition, we dichotomised age-groups and conducted analyses to assess the mean differences both for maternal and paternal age groups comparing a reference age group (25–29 years) with younger (≤ 20 years) and older (≥ 35 years) age groups. We found a statistically significant difference in children’s autistic-like symptoms between the maternal age groups (F_4, 5713_ = 5708, *p* < 0.001). A Tukey post hoc test revealed that the child autistic-like traits scores were significantly higher in younger age mothers (0.42 ± 0.28, *p* < 0.001) compared to the reference age group of 25–29 years old mothers. There was no statistically significant difference in child autistic-like traits scores between the reference age group and the older maternal age group (*p* = 0.249). For paternal age groups, a Tukey post hoc test showed that the child autistic-like traits were significantly higher only in the older age group (0.28 ± 0.21, *p* < 0.001) compared to the reference age group, after adjusting for sociodemographic variables. Detailed information on these analyses can be found in Additional file 1: Table.

## Discussion

Using a large population-based cohort of child development, we examined several key factors that could explain the association between maternal age at conception and autistic-like traits in offspring. These key factors included the effect of maternal mentalizing skills and maternal autistic-like traits. We found that the relationship between both maternal and paternal age best fits a U-shaped pattern (quadratic relationship) with child autistic-like traits. In addition, we found that maternal autistic-like traits predicted child autistic-like traits and that higher maternal mentalizing skills were associated with less maternal autistic-like traits. Although maternal age, autistic-like traits, and mentalizing skill were independently associated with child autistic-like traits, their associations were modest with small effect sizes (*f*^2 ^< 0.03). Our hypothesis that maternal autistic-like traits and mentalizing skills explained much of the relationship between increasing maternal age and child autistic-like traits in offspring was not met, as there was not a substantial change in the effect estimates of maternal age, and the relationship between maternal age and child autistic-like traits remained significant even when including these variables as covariates.

Our results support findings that maternal mentalizing skills are inversely associated with autistic-like traits in children [62–65]. Furthermore, we found that maternal autistic-like traits were also inversely associated with maternal mentalizing skills. Interestingly, the association between maternal age and child autistic-like traits was not linear, but formed a U-shaped distribution (Fig. 1). U-shaped relationships have been reported previously in clinical samples, although with less consistency [27, 66, 67]. Idring et al., (2014) reported that the effect of maternal age on autism in offspring was nonlinear, with the sharpest increase in likelihood after age 30, whereas the paternal age effect was linear, with higher ages increasing the likelihood. Sandin et al., (2016) also demonstrated a quadratic relationship between parental age (both maternal and paternal age) with an increased relative likelihood of autism. Interestingly, while our findings support a relationship with parental age and child autistic-like traits, our sensitivity analysis using a categorical approach based on age-categories supported by the literature showed that it is the younger maternal age group and the older paternal age group that appears to drive the nonlinear relationships. However, it is important to note that our categorical analyses have less power than our continuous analyses.

Prior research has explored the underlying mechanism between paternal age and a childhood diagnosis of autism. One frequently proposed explanation relates to the potential for increased rates of de novo mutations in older fathers [68, 69] and epigenetic modifications [70]. Our study also demonstrated that older paternal age had a stronger relationship with childhood autistic-like traits compared to older maternal age; however, the high covariance between maternal and paternal age makes this relationship difficult to disentangle. Whilst de novo mutation rates in fathers is one possible explanation, another is that parents with more autistic-like traits simply take longer to find a mate because sexual selection favors individuals with lower numbers of autistic-like traits. These two explanations are not mutually exclusive and need to be tested separately.

In our analysis, unexpectedly, younger maternal age was also associated with higher autistic-like traits in children. A potential explanation for this finding may relate to difficulties in reading social cues related to intimate relationships during a period of time where adolescents have greater risk-taking. Alternatively, it is possible that the women with higher levels of autistic-like traits may engage in more risk-taking behaviors during pregnancy, including exposures to drugs, smoking, or alcohol. Previous studies have shown that these environmental exposures are linked to a higher likelihood of having children within the autism spectrum [71–74]. Other potential factors associated with younger mothers, including prenatal complications, having been linked to an increased likelihood of later adverse outcomes [75]. It could also be that younger parental age reflects different mating behaviour in individuals with higher numbers of autistic-like traits, such as prioritising starting a family earlier, or a different attitude towards family planning. Finally, referral bias could also contribute to the finding that younger parents have children with higher rates of autistic-like traits, as younger parents were also underrepresented in our study, suggesting that we should be careful generalizing results for this group to the whole population.

## Strengths and limitations

The strengths of our study include the embedding within a large population-based cohort of child development, which included prospective follow-up that began in prenatal life. The study includes rigorous data and quality control processes and we were able to assess both maternal mentalizing skills and maternal autistic-like traits in a large sample of mothers.

However, our study also has some limitations. First, while we were able to collect data on both autistic-like traits and mentalizing skills in a large population of mothers, we did not collect this data in a large number of the fathers. Thus, we could not test whether the relationships we found in the mothers are also present in the fathers. Second, the reliability of the maternal mentalizing measure was low, as reported in our method section, therefore our measure may not have reliably captured mentalizing skills in the mothers. Thirdly, while there is a correlation between SRS scores and autism [76, 77], the SRS may be considered a better measure of social impairments rather than defining a child on the autism spectrum. In relation to maternal autistic-like traits captured by the AQ, while the SRS and AQ show a relatively strong correlation (0.64), they are different measures, as the AQ reflects autistic traits in the mother and the SRS autistic traits in the child. Although SRS and AQ are highly correlated, reflected in the content of their items (e.g., difficulty with changes in the routine, unable to understand the meaning of conversations), there are items in the SRS (e.g., playing with toys in a strange way, talking to people with an unusual tone) which are not included in the AQ. Further, it is possible that some of the strength of the correlation is likely related to shared rater bias, since the mother completed both questionnaires. Lastly, mothers who participated in this study tended to be older and of higher educational status compared to non-responders. As a result, mothers within the younger age group may have been underrepresented in the higher SRS scores.

## Conclusions

We found that parental age, and notably paternal age, is associated with higher autistic-like traits in offspring and the relationship best fits a U-shaped curve. Younger parental age was also associated with increased autistic-like traits in offspring, in particular in mothers. Further, higher rates of maternal autistic-like traits and lower performance on mentalizing tasks in mothers were associated with higher rates of autistic-like traits in offspring. Whereas it is possible that the relationship between older and younger mothers entails similar underlying mechanism, it is also possible that multiple, disparate mechanisms contribute to the tails of the U-shaped curve.

## Supplementary Information

**Additional file 1:** **Fig S1.** Flow chart of participants included for analyses. **Table S1.** Parental age groups and child autistic-like traits (*n* = 5718). **Table S2.** Pearson correlation coefficients among variables.** Fig S2.**Quadratic graphs between parental age and child autistic traits at 5/6 years-of-age.**Table S3.** Association between maternal age and child autistic-like traits among mothers with parity of first pregnancy (*N* = 5,061). **Fig S3.** Quadratic graphs between maternal age and child autistic-like traits after adjusting for maternal characteristics.** Fig S4.** Polynomial Regression. **Fig S5.** Spline Regression.

## Data Availability

The datasets analyzed during the current study are not publicly available due to the terms and conditions participants agree to when they participate in Generation R, but are available from the corresponding author on reasonable request.
