# Supplementary material for: Maternal age, autistic-like traits and mentalizing as predictors of child autistic-like traits in a population-based cohort
Source: Mol Autism. 2022 Jun 15;13:26. doi: 10.1186/s13229-022-00507-4 (PMC9199218; doi:10.1186/s13229-022-00507-4)

**Supplementary Figure 1**

*Flow chart of participants included for analyses.*

**7,254 participants enrolled**

**and gave consent to this study**

Missing data on maternal age

(*n =* 548)

**6,706 participants with available data**

**on maternal age**

Missing data on child autistic-like traits

(*n*= 988)

**5,718 participants with complete data**

**on maternal age and child autistic-like traits**

**Supplementary Table 1**

*Parental age groups and child autistic-like traits (N= 5,718)*

| **Maternal Age Groups** | ***N* = 5,718** | **Mean (SD)** | **Sig.** |
| --- | --- | --- | --- |
| Younger age group (< 20 years) | 167 | 0.42 (0.28) | <.001 |
| *Reference age group (25 - 29 years)* | *1470* | *0.29 (0.21)* | *-* |
| Older age group (> 35 years) | 1118 | 0.28 (0.24) | .249 |

*Note.*

After adjusting for confounders, the significance difference between younger age and reference age in maternal age group still appears.

| **Paternal Age Groups** | ***N* = 5,718** | **Mean (SD)** | **Sig.** |
| --- | --- | --- | --- |
| Younger age group (< 20 years) | 58 | 0.40 (0.32) | .028 |
| *Reference age group (25 - 29 years)* | *1085* | *0.32 (0.23)* | *-* |
| Older age group (> 35 years) | 2103 | 0.28 (0.21) | <.001 |

*Note.*

After adjusting for confounders, the significance difference between younger age and reference age in paternal age group disappeared.

**Supplementary Table 2** *Pearson correlation coefficients among variables.*

|  | 1. | 2. | 3. | 4. | 5. | 6. | 7. | 8. | 9. | 10. |  |
| --- | --- | --- | --- | --- | --- | --- | --- | --- | --- | --- | --- |
| 1. Child autistic-like traits |  |  |  |  |  |  |  |  |  |  |  |
| 2. Maternal age | **-.080** |  |  |  |  |  |  |  |  |  |  |
| 3. Paternal age | **-.067** | **.603** |  |  |  |  |  |  |  |  |  |
| 4. Maternal mentalizing skill | **-.092** | .024 | -.023 |  |  |  |  |  |  |  |  |
| 5. Maternal autistic-like traits | **.167** | **-.092** | -.024 | **-.152** |  |  |  |  |  |  |  |
| 6. Child age | **.042** | **-.054** | **-.032** | -.021 | .015 |  |  |  |  |  |  |
| 7. Child sex | **-.103** | -.011 | .006 | .000 | .008 | .005 |  |  |  |  |  |
| 8. Child national origin | **.049** | **-.077** | -.014 | **-.115** | **.155** | **.044** | .001 |  |  |  |  |
| 9. Maternal education | **-.113** | **.244** | **.112** | **.210** | **-.211** | -.025 | -.010 | **-.108** |  |  |  |
| 10. Paternal education | **-.100** | **.229** | **.103** | **.114** | **-.157** | **-.035** | -.006 | **-.106** | **.514** |  |  |

Bold denotes significant at *p* <.050.

**Supplementary Figure 2** *Quadratic graphs between parental age and child autistic traits at 5/6 years-of-age.*

**
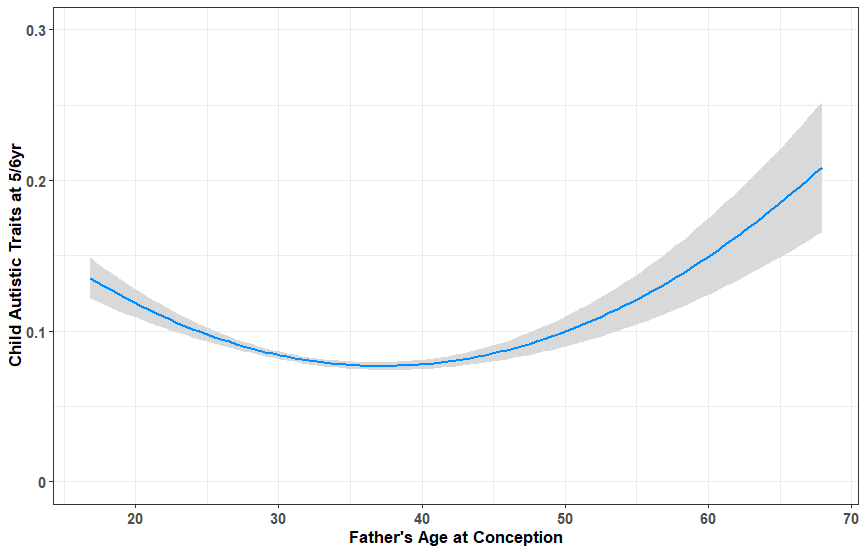

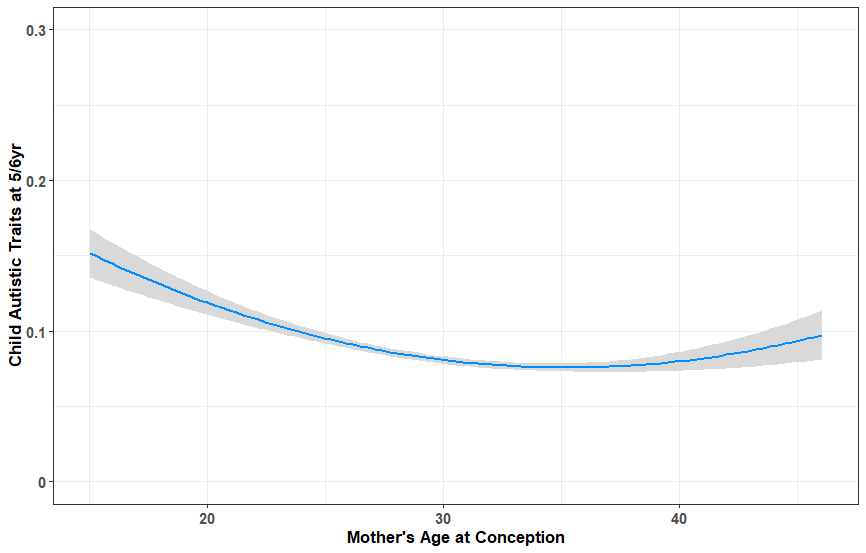
**

**Child autistic-like traits at 5/6 years-of-age**

**Child autistic-like traits at 5/6 years-of-age**

**Supplementary Table 3**

*Association between maternal age and child autistic-like traits among mothers with parity of first pregnancy (N= 5,061)*

|  | ***Model 1*** | ***R^2^***  ***(change)*** | ***Model 2*** | ***R^2^***  ***(change)*** | ***Model 3*** | ***R^2^***  ***(change)*** | ***Model 4^a^*** | ***R^2^***  ***(change)*** | ***Model 4^b^*** | ***R^2^***  ***(change)*** |
| --- | --- | --- | --- | --- | --- | --- | --- | --- | --- | --- |
|  | *β (95% CI)* | *p* | *β (95% CI)* | *p* | *β (95% CI)* | *p* | *β (95% CI)* | *p* | *β (95% CI)* | *p* |
| ***Step 1*** |  | **(.015)** |  |  |  |  |  |  |  |  |
| *Maternal Age* | -.116 (-.147; -.085) | <.001 | -.113 (-.143; -.082) | <.001 | -.069 (-.101; -.036) | <.001 | -.071 (-.103; -.039) | <.001 | -.062 (-.094; -.029) | <.001 |
| ***Step 2*** |  |  |  | **(.006)** |  |  |  |  |  |  |
| *Maternal Age Squared* |  |  | .080 ( .050; .111) | <.001 | .058 ( .027; .089) | <.001 | .056 ( .025; .087) | .001 | .053 ( .022; .083) | .001 |
| ***Step 3*** |  |  |  |  |  | **(.029)** |  |  |  |  |
| *Child Age* |  |  |  |  | .038 ( .007; .069) | .017 | .037 ( .006; .068) | .021 | .042 ( .009; .074) | .012 |
| *Child Sex* |  |  |  |  | -.210 (-.269; -.151) | <.001 | -.211 (-.269; -.152) | <.001 | -.212 (-.271; -.153) | <.001 |
| *Child National Origin* |  |  |  |  | .006 (-.002; .013) | .120 | .005 ( -.003; .002) | .226 | .001 ( -.006; .009) | .781 |
| *Maternal Education* |  |  |  |  | -.115 (-.146; -.083) | <.001 | -.101 (-.113; -.068) | <.001 | -.085 (-.117; -.052) | <.001 |
| ***Step 4^a^*** |  |  |  |  |  |  |  | **(.006)** |  |  |
| *Maternal Mentalizing Skills* |  |  |  |  |  |  | -.073 (-.117; -.029) | .002 | - | - |
| ***Step 4^b^*** |  |  |  |  |  |  |  |  |  | **(.023)** |
| *Maternal Autistic-like Traits* |  |  |  |  |  |  | - | - | .158 ( .121; .194) | <.001 |

*Note: R squared change is calculated by subtracting the changed explained variance in the model with the explained variance of the previous model*.

β = standardized beta, 95% CI = 95% confidence interval. Bold denotes significance ( *p* < 0.05)

*
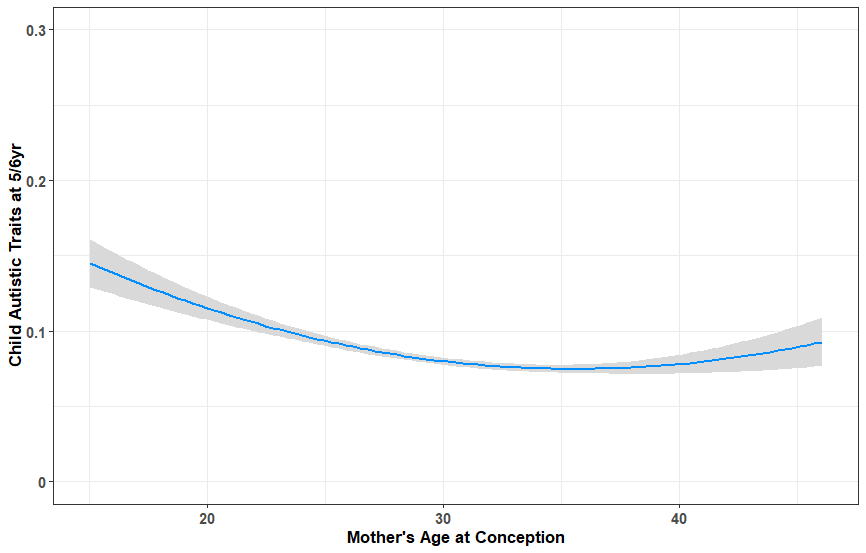
***Supplementary Figure 3** *Quadratic graphs between maternal age and child autistic-like traits after adjusting for maternal characteristics.*

*
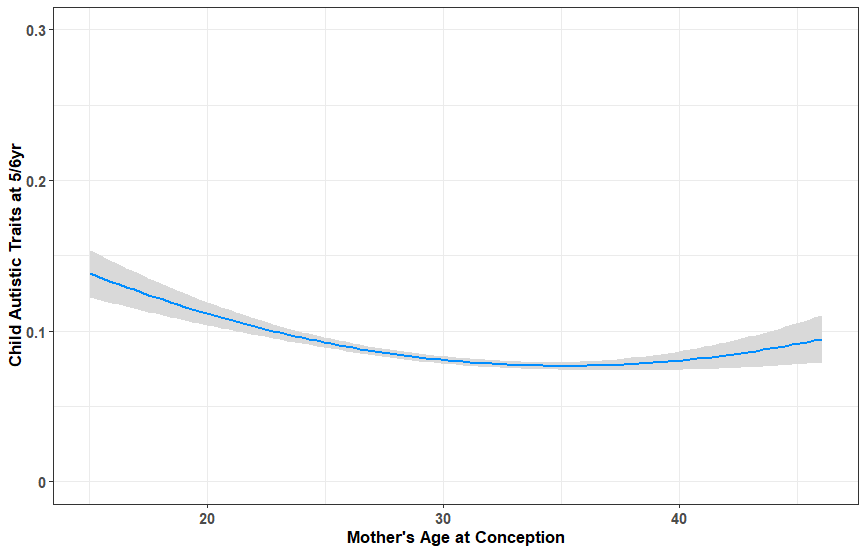
*

**Child autistic-like traits at 5/6 years-of-age**

**Child autistic-like traits at 5/6 years-of-age**

*Quadratic graph (child autistic traits at 5/6 years) after adjusting for maternal autistic-like traits Quadratic graph (child autistic traits at 5/6 years) after adjusting for maternal mentalizing skill*

*
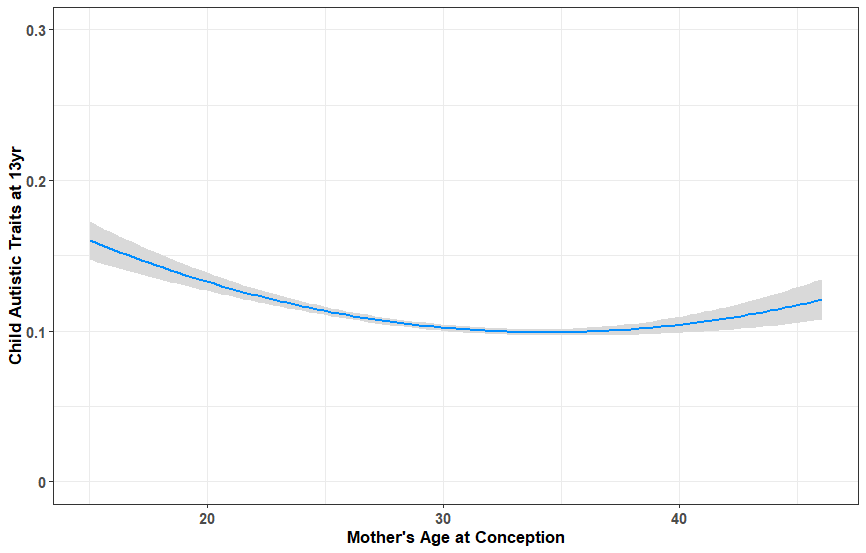

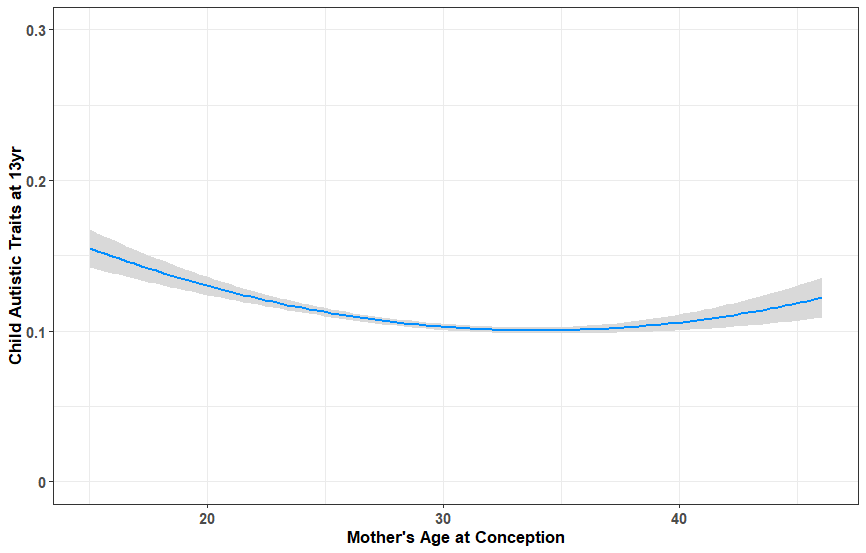
*

**Child autistic-like traits at 13 years-of-age**

**Child autistic-like traits at 13 years-of-age**

*Quadratic graph (child autistic traits at 13 years) after adjusting for maternal autistic-like traits Quadratic graph (child autistic traits at 13 years) after adjusting for maternal mentalizing skill*

*
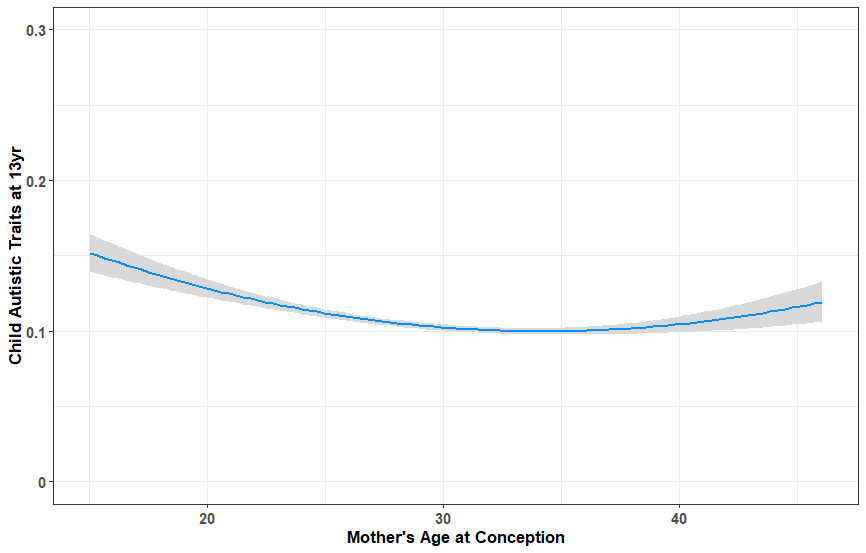

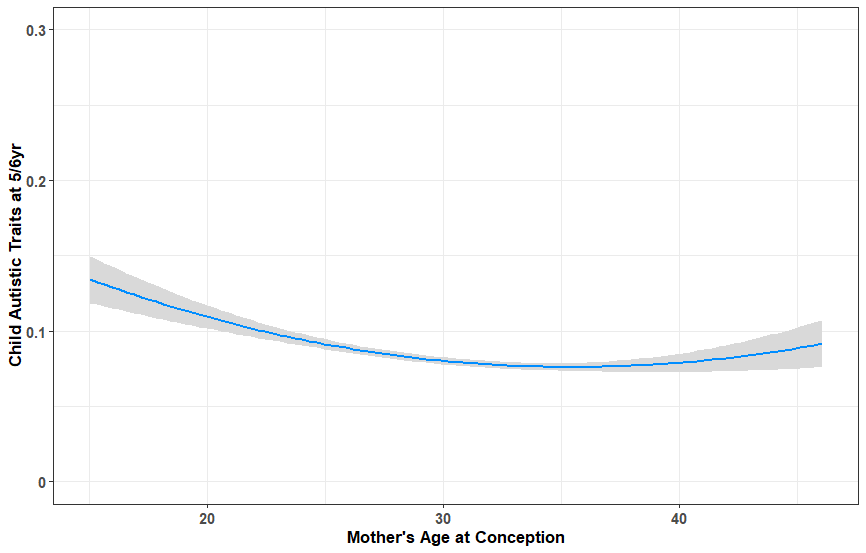
*

**Child autistic-like traits at 5/6 years-of-age**

**Child autistic-like traits at 13 years-of-age**

*Quadratic graph (5/6y) after adjusting for maternal autistic-like traits and mentalizing skills Quadratic graph (13y) after adjusting for maternal autistic-like traits and mentalizing skills*

**Supplementary Figure 4** *Polynomial regression*

*
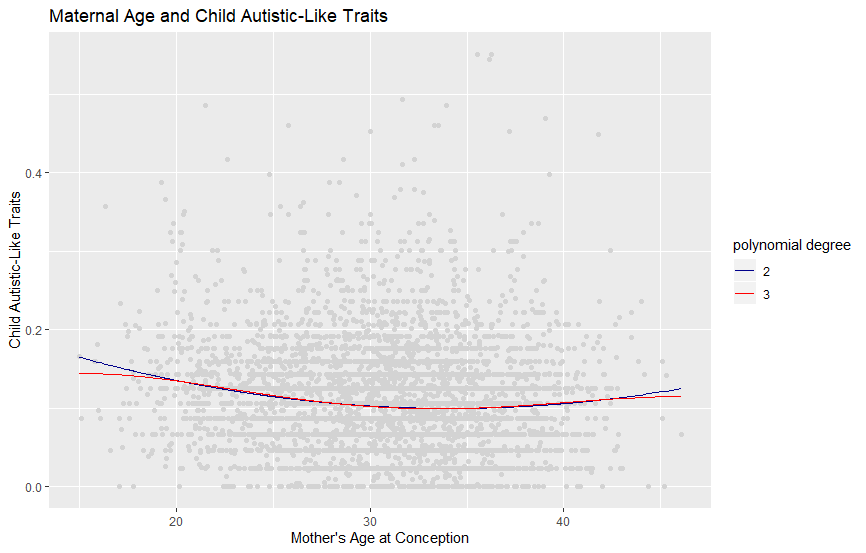
*

**Supplementary Figure 5** *Spline regression*


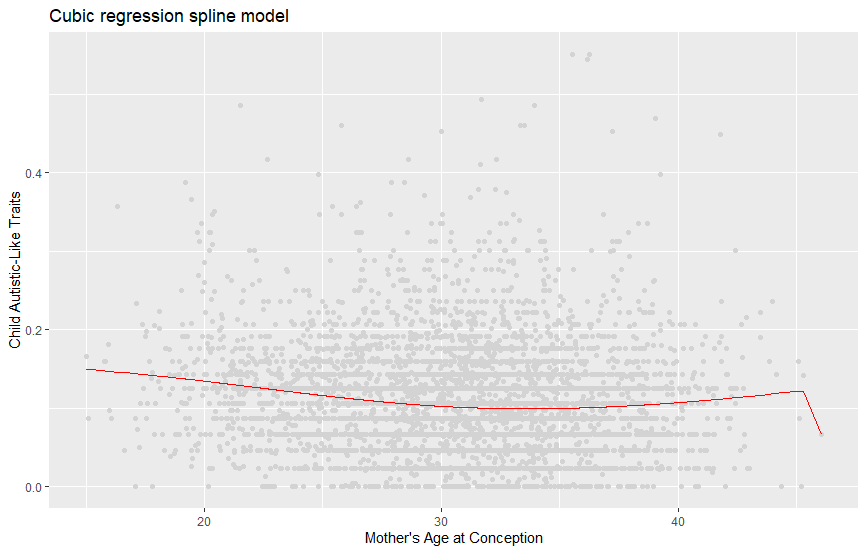

Supplement: Supplementary file 1 — Additional file 1: Fig S1. Flow chart of participants included for analyses. Table S1. Parental age groups and child autistic-like traits (n = 5718). Table S2. Pearson correlation coefficients among variables. Fig S2.Quadratic graphs between parental age and child autistic traits at 5/6 years-of-age.Table S3. Association between maternal age and child autistic-like traits among mothers with parity of first pregnancy (N = 5,061). Fig S3. Quadratic graphs between maternal age and child autistic-like traits after adjusting for maternal characteristics. Fig S4. Polynomial Regression. Fig S5. Spline Regression. [file 13229_2022_507_MOESM1_ESM.docx]
